# Resumption of Elective Surgery Following COVID-19 Outbreak, Guideline for Female Pelvic Medicine and Surgery 

**Published:** 2020-03

**Authors:** Zinat Ghanbari, Fatemeh Mostaan, Tahereh Eftekhar, Maryam Deldar, Nasrin Changiz, Khadijeh Adabi

**Affiliations:** 1 Division of Female Pelvic Medicine and Surgery, Department of Obstetrics and Gynecology, Imam Khomeini Hospital Complex, Tehran University of Medical Sciences, Tehran, Iran; 2 Maternal, Fetal and Neonatal Research Center, Tehran University of Medical Sciences, Tehran, Iran; 3 Maternal Health Department, Ministry of Health and Medical Education, Tehran, Iran

**Keywords:** Coronavirus, COVID-19, Elective Surgery, Female Pelvic Medicine

## Abstract

Division of Female Pelvic Medicine and Surgery, Department of Obstetrics and Gynecology, Emam Khomeini Hospital, Tehran University of medical sciences proposed a clinically relevant algorithm to guide appropriate decision making based on underlying risk stratification and resource utilization in order to resume elective surgeries, following COVID-19 pandemic crisis. The consequence of standardized decision-making factors and transparency of the principles will provide more assurance, consistency, and reliability on both sides, care providers and the patient. It also will decrease ethical dilemmas and moral criticism for surgeons. Eventually, this approach is applicable in any other disaster preparedness as a logical stratification of surgical indications for the female pelvic floor surgical procedures.

The World has experienced an unprecedented health emergency from the COVID-19 pandemic (1). In response to the COVID-19 pandemic, Iran’s Health ministry like many other authorities around the world such as the American College of Surgeons and the American Society of Anesthesiologists recommended postponing non-essential surgical procedures (2-5).

The rational for this advisory was the concern that elective procedures may lead to the spreading of the coronavirus within services and exhaust medical resources necessary to handle coronavirus epidemic (6). 

It is vital to classify various levels of necessary care, so surgical procedures are stratified into three categories based on its urgency state:

• Category 1, the most urgent, which should be approached within one month.

• Category 2 could be performed within three months.

• Category 3 could be postponed up to one year.

First category includes surgeries which if significantly delayed could cause major harm. Second and third categories are mainly elective procedures that could be delayed for extended time period. Elective procedures can practically be classified into “essential “, which implies the risk of increased adverse outcomes by postponing surgical care for an undetermined time period, contrasted with “non-essential “, which indicates purely elective surgeries that are not time-sensitive (7).

To guarantee the safety and feasibility of elective surgical procedures during the COVID-19 pandemic crisis, each hospital and surgical field should thoughtfully review all scheduled elective procedures with minimized adverse event. Categorization should be done by taking into account the specific circumstances of the patient including the extent of the pain and quality of life and the impact on daily activities if the surgery is deferred (3).

Pelvic floor disorders (PFD) include disorders of urinary and fecal incontinence, pelvic organ prolapses, and other lower urinary and bowel tract dysfunction (8). According to the evidence and expert opinion, pelvic floor surgical procedures are included in the non-essential category during the COVID-19 pandemic (4). Multidimensional conditions of Pelvic floor disorders (PFD) not only are prevalent but also disrupt wide aspects of quality of life, intimate and social relationships, and socioeconomic states of patients (8-10). The uncertainty on the predicted time course of COVID-19 outbreak and unintentional consequences imposed by postponing scheduled surgery may have immense reflection on patient’s quality of life (11).

It is important to take into consideration, many patients continue to have ongoing healthcare demands, but not essential which are currently being deferred due to the pandemic state. Health care providers including surgical services should be prepared to approach to the restrained surgical needs and prioritize cases in an appropriate time.

Relatively low and stable incidence of COVID-19 for at least 14 days should be considered to allow facilities to provide care for patients needing non-emergent procedures (12, 13). It is imperative to design and implement clinically relevant algorithms to guide appropriate decision making based on underlying risk stratification and resource utilization (5).

Division of female pelvic medicine and surgery, Department of Obstetrics and Gynecology, Emam Khomeini Hospital, Tehran University of medical sciences, which is a fellowship/residency training group, designed a set of principles and issues to help plan for resumption of elective surgical procedures. This is a committee consensus of Iranian Female Pelvic Medicine and surgery society and has been approved by health ministry. Table 1 provides the suggested stratification of surgical indications for the female pelvic floor surgical procedures.

This protocol has been designed according to the female pelvic medicine and surgery fellowship training curriculum and procedures requiring multidisciplinary services such as vesicovaginal fistula or rectovaginal fistula are not included.

**Table 1:** Suggested stratification of surgical indications for female pelvic floor surgical procedures

**Female pelvic floor surgeries**	**Non-Essential**	**Essential**	**Urgent-Elective**
A) Urologic surgeries including: TOT/TVT Pubovaginal sling Retropubic urethropexies (Burch-MMK) Intravesical botox injection		If: -Failure of conservative treatment -Patient's request -Progressive symptoms -Financial disability for conservative treatment + Stipulations 1&2	_
B) Pelvic organ prolapse Apical Prolapse beyond the hymen Anterior compartment prolapses beyond the hymen Posterior compartment prolapse beyond the hymen		Refer to algorithm (1) + Stipulations 1&2	Refer to algorithm (1) + Stipulations 1&2
C) Genital anomaly surgeries including: Mullerian anomalies		If: Hematometra/ Hematocolpus without severe abdominal pain as well as without acute urinary retention + Stipulations 1&2	If: Hematometra/ Hematocolpus with severe abdominal pain or with acute urinary retention + Stipulations 1&2
D) Perineal tears repair (3&4 grades)	(Chronic)	If impaired quality of life	_

Stipulation (1): Patient’s informed consent

Stipulation (2): Consultation with Forensic Medicine Specialists

Whenever effectiveness of non-surgical trials was not sufficient for PFDs, in order to estimate the necessity of surgical interventions, it is important to consider symptom severity from the patient’s perspective and also from clinician’s viewpoint, and evaluating the multifaceted impact of PFD on a women’s Quality of life (8, 14).

Some elective non-essential surgeries will turn into essential at some points in time, depending on how long this pandemic will prevail and considering the impact of PFD on a women’s Quality of life. In this regard, it is recommended to evaluate appropriate indications on a case-by-case basis. Figure 1 provides a decision-making algorithm based on elective surgical indications of the pelvic floor surgical procedures.

**Figure 1:**
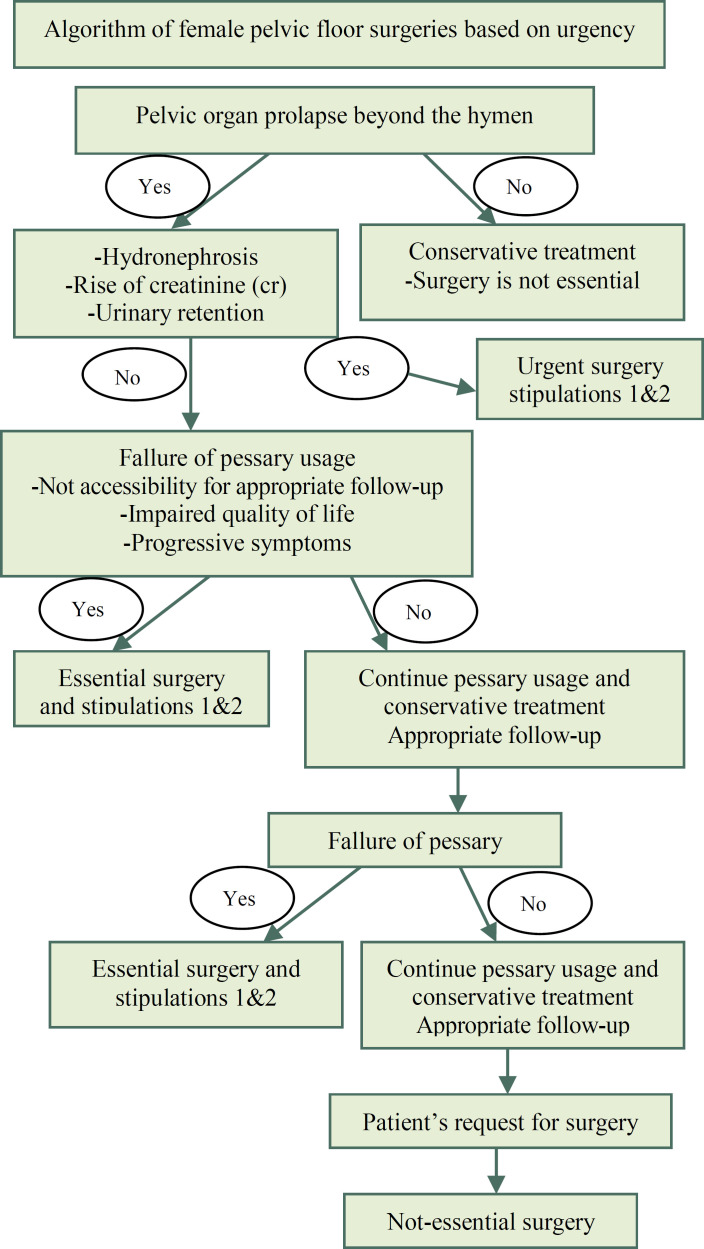
Decision-making algorithm based on elective surgical indications of pelvic floor surgical procedures

Patients may have numerous questions and concerns regarding the risks threatening their safety. Conclusive patient information and clear communication will be paramount (15). Consultation with forensic medical specialists and infectious diseases specialist is very important for safely scheduling elective care. 

In order to reopening of elective surgery, it is required to consider all aspects of care including adequate facilities for pre, intra and postoperative care, access to various blood products, estimated postoperative hospital stay length, need for postoperative ICU admission and whether personal protective equipment (PPE) and COVID-19 tests are available (16). Providers should prioritize surgical procedures considering patients past medical history and assess any risk factor predisposing to COVID-19 morbidity and mortality (17).

In consequence, standardized decision-making factors and transparency of the principles will provide more assurance, consistency and reliability on both sides, care providers and the patient. It also will decrease ethical dilemmas and moral criticism for surgeons. Eventually, this approach is applicable in any other disaster preparedness as a logical stratification of surgical indications for the female pelvic floor surgical procedures.
